# Mercury concentrations in store‐bought shrimp

**DOI:** 10.1002/fsn3.1659

**Published:** 2020-05-29

**Authors:** Angelle McCormick, Matthew D. Robertson, Rebecka Brasso, Stephen R. Midway

**Affiliations:** ^1^ St. Joseph's Academy Baton Rouge Louisiana USA; ^2^ Department of Oceanography and Coastal Sciences Louisiana State University Baton Rouge Louisiana USA; ^3^ Centre for Fisheries Ecosystems Research Fisheries and Marine Institute of Memorial University of Newfoundland St. John’s NL Canada; ^4^ Department of Zoology Weber State University Ogden Utah USA

**Keywords:** consumer, fat content, Hg exposure, penaeid shrimp, seafood

## Abstract

Most mercury exposure to humans comes from consumption of fish and shellfish; however, mercury concentrations are not known for all available seafood, particularly shrimp. Our objective was to estimate the concentration of mercury in a variety of store‐bought shrimp and then to compare total mercury concentrations to other information such as brand, harvest type, and total fat. We sampled a total of *n* = 159 shrimp from 10 different brands. Across 10 brands of shrimp, there was a significant effect of brand, with mean mercury concentrations among brands varying by up to an order of magnitude. We found no significant differences comparing shrimp between two capture types (wild‐caught and farm‐raised), which was perfectly collinear to whether shrimp were domestic or foreign. We did detect significant differences in mercury levels among different amounts of total fat in shrimp, with the lowest fat shrimp (1 g) having significantly more mercury than the highest fat shrimp (2 g). Although our results confirm that shrimp contains relatively low levels of mercury and is generally considered a good choice for consumers, this study is the first to report significant differences in mercury among both brands of shrimp and total fat content in shrimp.

## Graphical abstract

This study presents mercury concentrations in store‐bough (frozen) shrimp, which were all found to be low, but variable. No effect of harvest type or location was found, although total fat was related to mercury concentrations in shrimp.

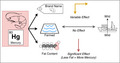

## 1 INTRODUCTION

The sole source of human exposure to methylmercury (the organic form of mercury) is consumption—most often in the form of fish, shellfish, and other aquatic protein sources (Clarkson, Magos, & Myers, 2003). Mercury exposure can result in various health risks to humans (Kim, Kabir, & Jahan, 2016) and has a wide range of ecological effects within ecosystems (Boening, 2000). The most commonly discussed adverse effects of mercury in humans are cognitive and developmental delays, primarily in children, and reproductive and neurological impairment (Díez, 2009). Mercury bioaccumulates in organisms’ tissues and biomagnifies in the food web, resulting in higher concentrations of mercury in higher trophic level species, which can also be increased with older aged individuals (McIntyre & Beauchamp, 2007). The risks of mercury toxicity have led various health agencies (e.g., the US Food and Drug Administration [FDA] and the World Health Organization [WHO]) to advise limits on the consumption of high‐trophic‐level fishes (Carrington & Bolger, 2002; WHO, 2006). However, understanding concentrations of mercury in low‐trophic‐level species is necessary because (a) lower trophic level species are still consumed by humans, (b) mercury concentrations may vary geographically with environmental factors, and (c) improved lower trophic level descriptions help us understand how toxicity may scale up throughout the food web (Lavoie, Jardine, Chumchal, Kidd, & Campbell, 2013).

Mercury uptake rates by low‐trophic‐level species are understudied, yet crucial for determining how mercury will biomagnify and accumulate throughout the food web. Mercury uptake can be influenced by various factors including the physiochemical environment (Ullrich, Tanton, & Abdrashitova, 2001), anthropogenic pollution (Carrasco, Díez, Soto, Catalan, & Bayona, 2008), and even biological factors, such as animal population density (Chen & Folt, 2005). For example, various studies have identified that methylmercury in marine ecosystems is mostly produced in low‐oxygen environments (Ullrich, Tanton, & Abdrashitova, 2001). Therefore, the transfer of methylmercury in the food web may be more pronounced in low‐oxygen environments like the deep ocean (Monteiro, Costa, Furness, & Santos, 1996) and coastal dead zones (Podar et al., 2015). As a result of the physical, chemical, and biological drivers that distribute mercury unevenly throughout the globe (Soerensen et al., 2010), geographical location has the potential to lead to wide variations in the amount of mercury available to accumulate in the food web (Driscoll, Mason, Chan, Jacob, & Pirrone, 2013). In order to provide accurate estimates of the potential risk of consuming low‐trophic‐level species, methylmercury measurements must be taken from a variety of environments and locations.

Shrimp are harvested throughout the globe (Gillett, 2008) and have the largest market share of any marine taxa in the United States (Groth, 2010). Low estimates of mercury concentrations in shrimp (0.012 ppm, wet weight [ww]; Smith & Guentzel, 2010) may suggest that shrimp should be consumed more often than other, higher trophic marine species. However, FDA estimates of mercury come from only two studies (Ache, Morse, & Kopfler, 2001; Hall, Zook, & Meaburn, 1978), neither of which collected any samples of imported or farmed shrimp, which currently comprise >90% of consumed shrimp in the United States (NMFS, 2018). Results from a recent meta‐analysis looking at mercury in shrimp found a significant discrepancy with the FDA estimate (US FDA, 2000), concluding that shrimp likely have a higher level of mercury than has previously been reported by government health agencies (Karimi, Fitzgerald, & Fisher, 2012). Should the current FDA estimate be inaccurate, advice centered around consuming greater quantities of shrimp may lead to adverse health effects. However, it is also worth noting that analytical techniques for measuring mercury have evolved over the decades, meaning that some earlier estimates cited in Karimi Fitzgerald and Fisher (2012) may have been estimated using different analytical techniques. For example, one recent study found that, based on per‐capita consumption rates, a slight increase in the mercury estimate of shrimp to 0.03 ppm would result in shrimp acting as the fourth largest contributor to mercury consumption in the United States (Sunderland, 2007). Furthermore, there are a large number of sources, producers, and sellers of shrimp available to most (U.S.) consumers, suggesting that if more were known about mercury concentrations in shrimp, consumers may be able to reduce their exposure through purchasing decisions.

The goal of this study was to estimate the concentration of mercury in a variety of store‐bought shrimp. Although we do not expect to find very high concentrations of mercury in shrimp, it remains important to quantify the variability in mercury among brands and also evaluate whether any attributes of shrimp correspond to higher mercury levels. Even if mercury levels are not high enough to be of concern to a general U.S. consumer, brand‐specific information may be relevant to high‐risk individuals (developing fetuses, young children, and those with chronic exposure through diet or occupation; Holmes, James, & Levy, 2009). Specifically, we sought to (a) estimate the mean and variability of mercury concentrations in different brands of shrimp, (b) to test the hypothesis that there is no difference in mercury between wild‐caught (domestic) shrimp and farm‐raised (foreign or imported) shrimp, and (c) correlate shrimp nutritional information to mercury concentrations. To our knowledge, there have been no direct comparisons of mercury concentrations between domestic wild‐caught shrimp and farm‐raised shrimp and very little investigation of mercury in shrimp overall.

## 2 MATERIALS AND METHODS

### 2.1 Tissue preparation

During April 2018, ten different brands of shrimp (one bag per brand) were purchased from large supermarkets in the Baton Rouge, LA, USA area. Although the purchases were somewhat opportunistic—that is, there were no specific brands targeted—limiting purchases to large supermarkets was intended to make sure that the brands we evaluated were common and widely available to regional consumers. All shrimp were purchased frozen and remained frozen until tissue processing. Prior to processing, information was recorded from the bags including *brand name*, *county of origin*, *count* (an industry code for size; e.g., colossal and extra jumbo), *harvest type* (wild‐caught or farm‐raised), *serving size*, and *total fat (g)*. Very few brands included a species scientific name, so no species‐level information was included in our analysis. From each bag, we selected approximately 15 individuals (depending on availability; larger shrimp sizes did not always result in 15 shrimp per bag) at random that were processed for mercury analysis, which included removal of the shell, intestinal tract, and any other material that was not white muscle. We saw no need to purchase multiple bags of shrimp for a single brand; for example, we did not need to purchase 15 bags of one brand, from which one shrimp per bag would have been sampled. Although a “bag” of shrimp may seem like an experimental unit or a group of nonindependent samples, we are confident of the following assumptions: (a) There is no effect of a bag (i.e., bags do not add or subtract mercury to samples), and (b) commercial shrimp is produced in a way that mixes catch across space and time, whereby a bag is a random sample from that brand. A small volume (approximately 2 g) of white muscle was removed from each individual, placed in a labeled cryovial, and stored in a freezer at −20°C. After the tissue froze, the cryovial lids were removed, Parafilm was placed over the tops of the vials, and a small hole was poked into the seal. Tissues were then dehydrated in a freeze dryer for 24 hr. After the samples were freeze‐dried, individual tissue samples were homogenized through pulverization using a clean mortar and pestle and the resulting dried tissue powder was placed back into a vial.

### 2.2 Mercury analysis

Because previously referenced mercury levels (e.g., EPA and FDA) are reported as total Hg and we know of no studies that report on percent methylmercury in shrimp, we conducted all our mercury measurements using total Hg. Approximately 20 mg of muscle (from the original 2 g sample) from each individual was loaded into a sample boat for total mercury analysis using a Nippon MA‐3000 Direct Mercury Analyzer. Each set of 20 samples was preceded by two samples of standard reference material (TORT‐3, lobster hepatopancreas, National Research Council Canada). Mean percent recovery for TORT‐3 was 100.3 ± 0.09% (*n* = 24) with relative significant differences in mercury concentrations <1%. Although mercury concentrations were determined in parts per million (ppm) dry weight (dw), we converted the concentrations to ppm wet weight (ww). This was done because the vast majority of published studies on mercury in shrimp report mercury concentrations in wet weight, and we wanted our numbers to be directly comparable. In order to convert our dry weights to wet weights, we used the following equation.

$$Hg_{ppm\left(\mathrm{ww}\right)}=\frac{100-75}{100}\times Hg_{ppm\left(\mathrm{dw}\right)}$$

where the estimated wet weight, Hg_ppm(ww)_, is a function of the dry weight, Hg_ppm(dw)_, multiplied by a proportion of water loss. In this case, we used a water loss constant of 75% that has been supported in a number of studies for use in shrimp (Campbell, Verburg, Dixon, & Hecky, 2008; Lavoie et al., 2010; Mortazavi & Sharifian, 2011).

### 2.3 Statistical analysis

We used general linear models for all analyses. For the analysis of mercury concentrations (Hg [ppm]) by brand, we used a 1‐way ANOVA, and for the analysis of harvest, we used a Welch's *t*‐test because we were only comparing two groups. For the analysis of total fat (g), we could have used a simple linear regression because total fat (g) is a continuous variable; however, we opted to use an ANOVA because (a) we were not sure if the trend would be linear, and (b) with only three unique values for total fat (g), it was appropriate to consider fat content as categorical. Further, an ANOVA would also allow us to test for any differences among total fat (g) groups, which a linear model would not do. With that being said, we recognize that a linear model could be used and, in a situation where there are more unique values of total fat (g), a linear regression might be a better model. All models used an a priori significance level of *α* = 0.05, and any significant effects were further evaluated with a Tukey's honestly significant difference (HSD) test to examine which pairwise comparisons differed.

## 3 RESULTS

We sampled a total of *n* = 159 shrimp from 10 different brands. The median number of individual shrimps per brand was 16, but ranged from only 14 to 18. Mercury concentrations across all shrimp from all brands were approximately normally distributed, though bounded by 0 on the left and with a few higher concentration samples skewing the distribution on the right. The mean (± standard deviation) of mercury concentration for all shrimp sampled was 0.02 ± 0.01 ppm ww. Three of the brands were farm‐raised, each from three different countries: Indonesia, Thailand, and Vietnam. The other seven brands were all wild‐caught from US waters. Harvest type (wild‐caught or farm‐raised) was confounded with being US domestic or foreign; all US domestic brands were wild‐caught, and all foreign shrimp were farm‐raised. For this reason, we considered these factors together because their collinearity would not provide any new results if analyzed separately. We also recorded seven different counts (sizes) of shrimp and three different amounts of total fat (1, 1.5, and 2 g per 4 ounce serving), which was the only nutritional information that varied across brands.

### 3.1 Mercury concentrations by brand, harvest, and fat content

**FIGURE 1:**
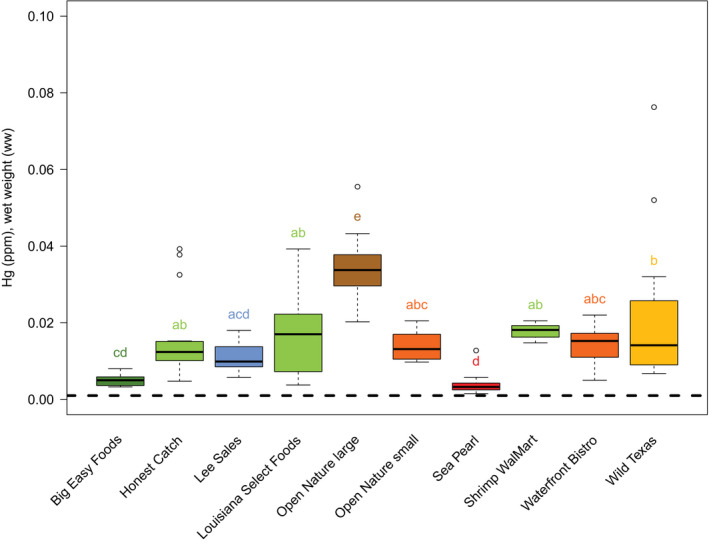
Box plots of mercury concentration by brand of shrimp. For each box plot, the box represents the interquartile range (IQR), the thick black line represents the median value, and the whiskers extend to 1.5 times the IQR. Outliers are represented by open circles. Letters above the box plots represent groupings based on Tukey's HSD *post hoc* multiple comparison test, where brands sharing the same letters (either individual letter or multiple letters) are not statistically different from each other. Box colors correspond to their grouping of letters, although boxes with different colors are not necessarily statistically different. The dashed black line (*y* = 0.001) represents the median mercury [ppm] in shrimp based on EPA data from 1990 to 2012 (Ache, Boyle, & Morse, 2000)

**FIGURE 2:**
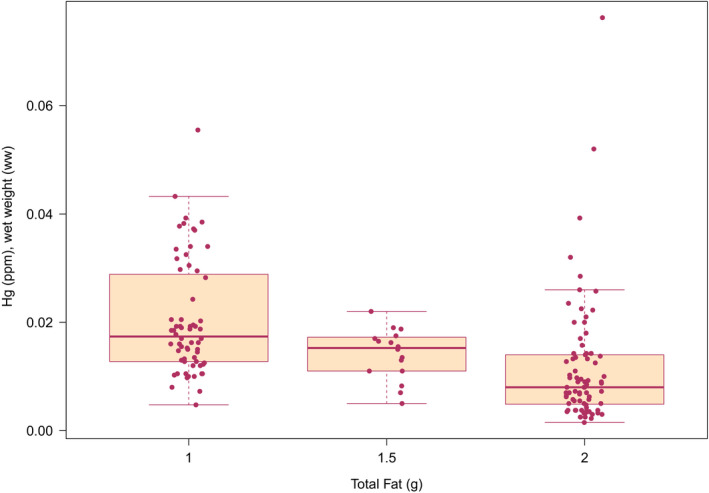
Box plots of mercury concentrations by amount of total fat (g) in shrimp from 10 different brands. Box plot descriptions can be found in the caption for Figure 1. Tukey's HSD test found a statistically significant difference in mercury concentration between shrimp with 1 g total fat and shrimp with 2 g total fat

Mean mercury for each of 10 different brands varied significantly (*F* = 16.7; *p* < .01; Figure 1). Open Nature Large brand had the highest mean mercury at 0.03 ± 0.01 ppm, while the Sea Pearl brand had the lowest mean mercury at 0.004 ± 0.003 ppm—a full order of magnitude lower. Most brands had mean mercury concentrations around 0.02 ppm. As stated above, harvest type (wild‐caught or farm‐raised) was confounded with US domestic or foreign shrimp, and therefore, our analysis effectively compares US wild‐caught shrimp to foreign farm‐raised shrimp. Although US wild‐caught shrimp had marginally lower mean Hg (0.015 ± 0.013 ppm) than foreign farm‐raised shrimp (0.016 ± 0.007 ppm), a Welch's *t*‐test found no significant difference between the US wild‐caught shrimp and foreign farm‐raised shrimp (*t* = 0.45, *p* = .65). An analysis of mercury by shrimp size (i.e., count) was not conducted because there were several size classes that contained only one brand, making any such analysis of size confounded to that of brand. We did analyze mercury concentration by total fat (g) and found that there was a significant effect of total grams of fat (*F* = 12.19; *p* < .01; Figure 2). Shrimp with 1 g of total fat had the highest mean mercury concentration, with mercury concentration decreased with increasing total fat (Figure 2). Shrimp with 1 g total fat had significantly higher mercury than shrimp with 2 g total fat (*p* < .01).

## 4 DISCUSSION

Overall, mercury concentrations in shrimp were relatively low and well below the Food and Drug Administration's (FDA) 1 ppm action level (US FDA, 2000). The mercury concentrations in the shrimp we sampled were much closer to (and still below) the values of fish designated as “bottom feeders” (≈0.1 ppm ww; Bahnick, Sauer, Butterworth, & Kuehl, 1994) and well below values reported for higher trophic level fishes (0.3–0.7; Cladis, Kleiner, & Santerre, 2014; Kidwell, Phillips, & Birchard, 1995; NESCAUM, 1998). Based on the maximum mercury we measured (0.08 ppm ww), the Environmental Protection Agency (EPA) would place shrimp in the “best choice” category and suggest that up to three meals per week are safe. Despite this overall good news of the shrimp being safe for consumption, we found several brands with higher levels of mercury in shrimp than had previously been reported. Specifically, Smith and Guentzel (2010) reported a value of measured mercury in shrimp to be 0.012 ppm ww. Although many brands we evaluated were around 0.012 ppm ww, several brands had mean levels and ranges that were above 0.012. We did not test any brands with mean mercury concentrations as high as those reported in Karimi Fitzgerald and Fisher (2012), who estimated a mean of 0.053 ppm ww; however, we did find individual shrimp that were at or above 0.05 ppm ww.

We detected an order of magnitude difference in mercury concentrations among several brands of store‐bought shrimp. Although all brands could be considered safe to eat according to the US FDA, our work suggests that consumption of different brands of shrimp could result in different mercury exposures. However, we found no significant differences in mercury concentrations between US wild‐caught shrimp and foreign farm‐raised shrimp. We did see an increase in mercury with decreasing total fat and a significant difference between shrimp with 1 g of total fat and shrimp with 2 g of total fat. This could be attributed to mercury's association with protein, and shrimp with greater total fat could have less protein by percent body mass.

We recognize the limitations of our study. First, although we sampled brands available at multiple large supermarkets in an urban area, the 10 brands we evaluated are only a small sample of shrimp that may be available nationwide. We have no data to suggest how well our 10 brands represent all consumer brands of shrimp. Another limitation was that we had little information on the timing and location of shrimp harvest. We assume that any mercury in the shrimp is imparted by the shrimp's environment while it is living, and that no mercury is transmitted to the shrimp after capture. Although we have country of origin information and we assume that the shrimp were captured and processed within the last several months, we have no specific or useable data on exactly when and where shrimp were harvested. As such, we are left to examine the large‐scale variables that we present in this study. Finally, it is known that crustaceans detoxify Hg through molting of the exoskeleton (Bergey & Weis, 2007; Keteles & Fleeger, 2001), yet we have no way of knowing the timing of molt for the individual shrimp in our study and thus no way to quantify the magnitude or variability of this potential effect.

In this study, we tested the idea that readily available shrimp contain equal amounts of mercury. Although all shrimp we studied had low levels of mercury, we did find substantial differences among brands, highlighting the fact that mercury exposure from shrimp may be more variable than previously thought. Even without brand information, simple nutritional facts—like total fat—can be useful information toward selecting for shrimp that minimize mercury exposure.

## CONFLICT OF INTEREST

The authors declare no conflict of interest before, during, and after this study.
